# AgoArmet and AgoC002: key effector proteins in cotton aphids host adaptation

**DOI:** 10.3389/fpls.2024.1500834

**Published:** 2024-11-28

**Authors:** Hui Xue, Mengjie Yan, Xiangzhen Zhu, Li Wang, Lizhen Chen, Junyu Luo, Jinjie Cui, Xueke Gao

**Affiliations:** ^1^ Research Base of Zhengzhou University, State Key Laboratory of Cotton Bio-breeding and Integrated Utilization, Institute of Cotton Research, Chinese Academy of Agricultural Sciences, Anyang, China; ^2^ State Key Laboratory of Cotton Bio-Breeding and Integrated Utilization, School of Agricultural Sciences, Zhengzhou University, Zhengzhou, China; ^3^ College of Plant Science and Technology, Huazhong Agricultural University, Wuhan, China

**Keywords:** aphid, salivary protein, Armet, C002, plant defense, VIGS

## Abstract

Aphids are insects that feed on phloem and introduce effector proteins into plant cells through saliva. These effector proteins are key in regulating host plant defense and enhancing aphid host adaptation. We identified these salivary proteins in the cotton aphids genome and named them AgoArmet and AgoC002. Multiple sequence alignment, protein structure analysis, and phylogenetic analysis of these proteins with related proteins from other insects showed that AgoArmet and Armet of *Aphis craccivora* have high sequence identity (97%) and belong to the same evolutionary branch and that AgoC002 shares the highest sequence identity (80%) and closest evolutionary relationship with C002 of *Aphis glyceins*. Expression profiling of AgoArmet and AgoC002 showed that they were most highly expressed in cotton aphids during the adult-3d period. Cotton aphids transferred to zucchini leaves resulted in a significant increase in the expression of *AgoArmet* and *AgoC002* within 48h. To investigate the functions of *AgoArmet* and *AgoC002*, we decreased the expression of these genes in cotton using virus-induced gene silencing (VIGS), which ultimately led to a 38% and 26% decrease in cotton aphids fecundity, respectively. Moreover, the reduction in *AgoC002* expression resulted in a significant (24%) reduction in body weight. Taken together, our findings demonstrate that AgoArmet and AgoC002 are key effector proteins involved in cotton aphids feeding and host adaptation.

## 1 Introduction

In nature, there is long-term co-evolution between plants and insects, and this “arms race” has led to the evolution of complex defense strategies employed by plants against herbivores, including constitutive and inducible defenses (Wu and Baldwin, 2010). To adapt to their host plants, insects have evolved different feeding styles and behaviors. For example, while feeding on a plant, phloem-feeding insects secrete saliva containing salivary proteins that inhibit plant defense responses (Tjallingii, 2006; Hogenhout and Bos, 2011; Elzinga and Jander, 2013; Liu et al., 2019). Currently, the known salivary proteins are divided into two main categories: excitons and effectors. Excitons are similar to pathogen-associated molecular patterns (PAMPs) and can be recognized by pattern-recognition receptors on plants and trigger PAMP-triggered immunity, or they can be recognized by plant resistance proteins, activating stronger effector-triggered immunity (Jones and Dangl, 2006; Rodriguez and Bos, 2013; Snoeck et al., 2022). For example, overexpression of *Myzus persicae* (peach aphid) salivary proteins Mp10, Mp42, and Mp58 in tobacco leads to a strong plant defense responses, causing traits such as leaf yellowing and cellular necrosis, and decreasing peach aphid fecundity (Rodriguez et al., 2014). Different from exictons, effectors, promote host susceptibility by interfering with various aspects of plant defense (Hogenhout and Bos, 2011), such as the activation of defense-related genes, the production of defensive compounds, and the reinforcement of physical barriers. For example, the *Helicoverpa armigera* salivary effector HARP1 directly interacts with the jasmonate ZIM-domain (JAZ) repressor and prevents coronatine insensitive 1-mediated JAZ degradation, thereby blocking jasmonic acid (JA) signaling (Chen et al., 2019). The salivary effector Bt56 promotes whitefly acclimation by stimulating the salicylic acid (SA) signaling pathway (Xu et al., 2019). Despite the importance of insect salivary proteins in plant-insect interactions, our understanding of their functions and mechanisms of action remains limited.

*Aphis gossypii* Glover (cotton aphid) belongs to the order Hemiptera, family Aphididae; these insects with piercing-sucking mouthparts are widely distributed in tropical, subtropical, and temperate regions and have become worldwide pests of cotton, a wide range of vegetables, and ornamental plants (Zhang et al., 2022a). Cotton aphids have adapted to several host plants via prolonged synergistic evolution by altering the composition and components of their saliva. Aphid salivary proteins enter the plant through the stinging mouthparts, and these salivary proteins can act as effectors to inhibit the defense responses of the plant; for example, Ca^2+^-binding proteins inhibit defense responses by binding Ca^2+^ within the plant (Will et al., 2007). In addition aphid saliva contains detoxifying effectors such as polyphenol oxidase, which oxidizes phenolics in plants and reduces their toxicity (Urbanska et al., 1998). Therefore, salivary proteins are crucial for the survival of aphids.

Currently, many salivary proteins including C002, Armet, Mp55, Me47, MIF, and DNase II have been identified in aphids and planthoppers (Mutti et al., 2008; Elzinga et al., 2014; Naessens et al., 2015; Wang et al., 2015; Kettles and Kaloshian, 2016; Huang et al., 2019). Armet has a dual function in mammals, functioning intracellularly as a component of the unfolded protein response in the lumen of the endoplasmic reticulum (ER) and extracellularly as a neurotrophic factor (Mizobuchi et al., 2007; Apostolou et al., 2008). Because of its second role, Armet is commonly referred to as midbrain astrocyte-derived neurotrophic factor (Petrova et al., 2003; Palgi et al., 2009). Armet is a highly conserved protein that may have conserved functions in mammals and insects and it was first discovered as an aphid effector protein by Wang in 2015 (Wang et al., 2015). Aphid Armet-induced SA accumulation in plants causes resistance to bacterial pathogens but not to aphids, enabling aphids to avoid bacterial infections and thereby promoting feeding, growth, and development (Cui et al., 2019). Recent studies have shown that Armet can inhibit plant defense responses and promote whitefly adaptation to tobacco by interacting with the tobacco cysteine protease inhibitor A-like protein NtCYS6 (Du et al., 2022). C002 is also an important salivary effector protein of aphids that is essential for aphid feeding and survival. C002 was found to be highly expressed in the salivary glands of *Acyrthosiphon pisum*, and inhibition of C002 gene expression caused massive mortality of *A*. *pisum* after feeding on pea plants; interestingly, the artificial feed-induced reduction in C002 expression did not significantly differ in mortality compared to normal-growing *A*. pisum, suggesting that C002 plays an important role in the mutualistic relationship between the insect and the host plant (Mutti et al., 2006). Another study found that silencing of C002 in *A*. *pisum* reduced stinging behavior and feeding time in host plants (Mutti et al., 2008). Bos et al. found that overexpressing the *MpC002* gene in *M*. *persicae* significantly increased the number of offspring (Bos et al., 2010). Because Armet and C002 are critical in aphid feeding and interaction with host plants, it is not clear whether Armet and C002 may also play important roles in cotton aphids.

In our study, we identified the Armet and C002 proteins in the cotton aphids genome (Zhang et al., 2022a) (*AgoArmet* and *AgoC002*, respectively) and analyzed their structures and evolutionary relationships with other insects Armet and C002 proteins. We also determined expression patterns of *AgoArmet* and *AgoC002* in different tissues at different developmental periods using reverse-transcription quantitative PCR (RT-qPCR). Finally, we transfected cotton plants with interference vectors targeting *AgoArmet* and *AgoC002* using virus-induced gene silencing (VIGS) technology and found that the reduction of *AgoArmet* and *AgoC002* expression significantly reduced cotton aphids acclimatization to cotton. The current pesticide control method of aphid control has many drawbacks. Our findings add to the understanding of the role of insect salivary proteins and contribute to the development of new crop protection strategies that target specific salivary proteins to enhance plant resistance to herbivorous insects.

## 2 Materials and methods

### 2.1 Insects

All *A. gossypii* insects used in this study were collected in 2015 from the experimental field of the Institute of Cotton Research, Chinese Academy of Agricultural Sciences (CAAS), and were kept in indoor incubators under the following conditions: 25 ± 1°C and 60% ± 2% relative humidity with a 14-h light:10-h dark photoperiod. *A*. *gossypii* was maintained on transgenic-free cotton.

### 2.2 Sequence analysis

**Figure 1:**
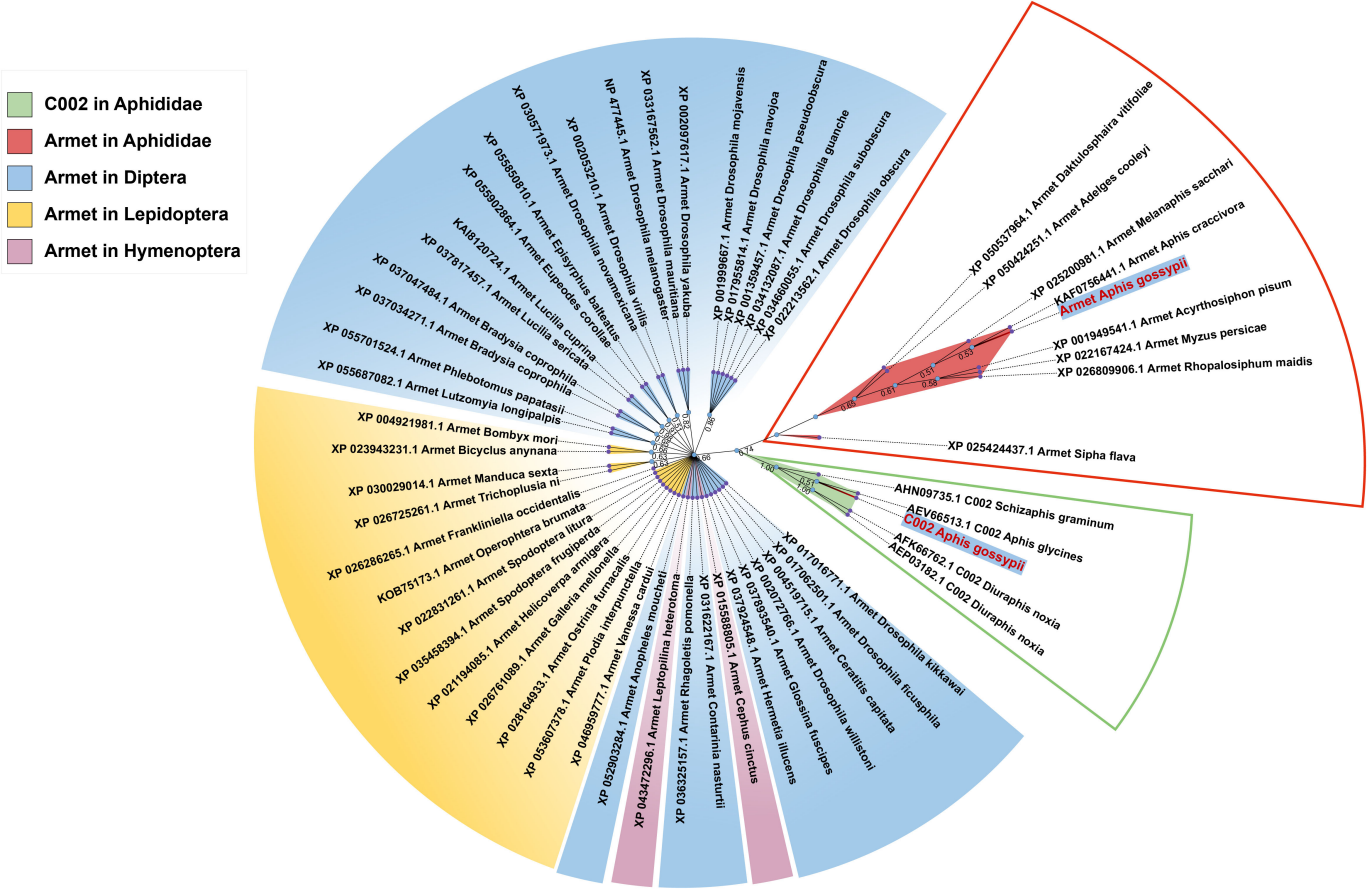
Phylogenetic analysis of Armet and C002 proteins from different insect orders. The names of proteins in the phylogenetic tree consist of the accession number of the sequence, gene name, and Latin species name. The red box indicates the evolutionary branch of Armet in Aphididae, and the green box indicates the evolutionary branch of C002 in Aphididae. Blue, yellow, and purple modules indicate the evolutionary branches of Armet in Diptera, Lepidoptera and Hymenoptera, respectively.

Nucleotide sequences of *AgoArmet* and *AgoC002* were obtained from the *A*. *gossypii* genome (Zhang et al., 2022a). The ExPASy translate tool (http://web.expasy.org/translate/) was used to deduce the amino acid sequences of *AgoArmet* and *AgoC002*. Further confirmation of sequence definitions was performed using the NCBI Blastn and Blastp modules. The isoelectric point and molecular mass were predicted using online tools (https://web.expasy.org/compute_pi/). Signal peptides were predicted using SignalP4.1 online software (https://services.healthtech.dtu.dk/services/SignalP-5.0/). The SWISS-MODEL online analysis tool (https://swissmodel.expasy.org/interactive) was used to predict the protein tertiary structure. A phylogenetic tree was constructed using the neighbor-joining method in MEGA version 7.0 (Kumar et al., 2016), and the GenBank accession unmber of amino acid sequences was shown in **Figure 1**. Statistical support for the tree was generated using bootstrap analysis with 1000 replicates. Multiple sequence alignment was performed using DNAMAN version 6.0.

### 2.3 RNA extraction and cDNA synthesis

Total RNA from *A. gossypii* samples was extracted using the TRIzol total RNA isolation system (Invitrogen, Carlsbad, CA, USA) according to the manufacturer’s protocol. RNA quality was evaluated on 1.0% agarose gels, and RNA was quantified using a Nano-Drop 2000 (Thermo Scientific, Wilmington, DE, USA). cDNA was synthesized from 1 μg of RNA using the PrimeScript RT Reagent Kit with gDNA Eraser (Takara, Dalian, China) according to the manufacturer’s protocol.

### 2.4 Expression profiles of *AgoArmet* and *AgoC002*

The head, thorax, and abdomen of cotton aphids at the 3-day-old nymph (3d-nymph), 1-day-old adult (1d-adult), and 3-day-old adult (3d-adult) were collected for profiling of *AgoArmet* and *AgoC002* expression. For host transfer analysis, we transferred second instar cotton aphids with cotton as the host to zucchini leaves, and collected samples at 12h, 24h, 48h, and 72h after transfer, while cotton aphids of the same period of time growing on cotton leaves were used as the control group. Three biological replicates were set up for each subgroup in each period, and 20 cotton aphids were collected in each biological replicate. RNA extraction and cDNA synthesis were performed as described in Section 2.3, and the cDNA was used as the template for RT-qPCR, which was performed with the StepOnePlus™ RealTime PCR System (Applied Biosystems, Foster City, CA, USA) using TransStart Top Green qPCR SuperMix (TransGen Biotech, China) in a 10 μl reaction volume under the following reaction conditions: 95 °C for 3 min followed by 40 cycles of 95 °C for 5 s and 60 °C for 30 s. *Dimethyladenosine transferase* (*DIMT*, GenBank: KM507111) and *peptidyl-prolyl cis-trans isomerase* (*PPCTI*, GenBank: KF018924) were used as reference genes for gene expression normalization (Zhang et al., 2015). Gene expression was calculated using the 2^− ΔΔCt^ method (Pfaffl, 2001). Primer information is shown in **Table 1**.

**Table 1:** Primer information.

Primer name	Primer sequence (5’-3’)	Regression equation	Amplification efficiency/%	Correlation coefficient (R^2^)
RT-qPCR				
Amret	F:GTGAAATGTCCAAACCTTTATC	y = -3.6125x + 34.795	89.2	0.998
	R:TATCGCACACTTGAGCAT			
C002	F:CCGATTAGCCAGAGTGTT	y = -3.4122x + 32.817	96.4	0.999
	R:TGGAAGGAGTGTTGGTAAG			
DIMT	F:AGCCACTAACCTAACATC	y = -3.5452x + 36.646	91.5	0.997
	R:TCAAGGAATGGTAGAGAAG			
PPCTI	F:GTCTTCATTACAGTCTATGG	y = -3.3621x + 29.922	98.4	0.997
	R:ATTGAGTGGTAGATGAGTT			
RNAi				
RNAi_Armet	F: CGACGACAAGACCGTG ATTTCAAGCACAATCCCGAA	/		
	R: GAGGAGAAGAGCCGTCG TATCGCACACTTGAGCATCC			
RNAi_C002	F: CGACGACAAGACCGTG AAGCTAGTTGTGCTGGTGGG	/		
	R: GAGGAGAAGAGCCGTCG TCTGCTTGATCATTTGCTGG			

### 2.5 Construction of VIGS cotton plants

We collected normally growing cotton aphids in the laboratory as samples for RNA extraction and cDNA synthesis. PCR amplification of the target gene was performed in a reaction consisting of 25 μl PrimeSTAR Max Premix (Takara, China), 1 μl forward primer, 1 μl reverse primer, 1 μl cDNA, and 22 μl RNase-free water at 98°C for 5 min; 35 cycles of 98°C for 10 s, 55°C for 15 s, and 72°C for 1 min; and 72°C for 7 min. Primer information is shown in **Table 1**. PCR products were evaluated by 1.0% agarose gel electrophoresis and purified using the Wizard^®^ SV Gel and PCR Clean-Up System (Promega, China) following the manufacturer’s protocol. The purified PCR product was mixed with 0.5 μl 10× T4 DNA Polymerase Buffer, 0.5 μl 0.1% bovine serum albumin (BSA), 0.25 μl 1.7 mM dNTP Mixture, 0.1 μl T4 DNA polymerase, 2.5 μl PCR product, and 1.15 μl RNA-free water (reaction solution I). The pTRV2 vector, which was kindly donated by Prof. Liu Yule from Tsinghua University, was digested with Pst I (Takara, China) according to the manufacturer’s protocol, and the digested vector was purified using the Wizard^®^ SV Gel and PCR Clean-Up System (Promega, China). The purified vector was mixed in a 1:1 ratio with 2 μl 10× T4 DNA Polymerase Buffer, 2 μl 0.1% BSA, 1 μl 1.7 mM dNTP Mixture, 0.4 μl T4 DNA polymerase, and 14.6 μl RNA-free water to obtain reaction solution II. Reaction solutions I and II were incubated at 37°C for 30 min, 75°C for 20 min, and 70°C for 3 min; finally, reaction solutions I and II were mixed in a 1:1 ratio and incubated at 22°C for 10 min to obtain the ligation product. The ligation product was then used for the construction and detection of positive recombinants following the manufacturer’s protocol of Trans1-T1 Phage Resistant (TransGen Biotech, China), and plasmid extraction was performed using the TIANprep Mini Plasmid Kit (Tiangen Biotech, China).

The plasmid (2.5 μl) was mixed with 100 μl of an *Agrobacterium tumefaciens* culture, placed in an ice bath for 30 min, flash frozen in liquid nitrogen for 2 min, heat-treated for 90 s at 37°C, and placed in an ice bath for 5 min. Then 600 μl of antibiotic-free LB liquid medium was added and cells were incubated at 28°C, 190 rpm for 4 h. The bacterial culture (200 μl) was then spread on LB solid medium containing kanamycin (50 μg/ml), and rifampicin (25 μg/ml), and incubated inverted at 28 °C until colonies were visible (48 h). Next, single colonies were picked and placed in 1 ml of LB liquid medium, incubated until the bacterial solution became turbid and OD = 1.0. pTRV1 vector was mixed with pTRV2 or pTRV2-Gene in a 1:1 ratio. After centrifugation at 3000 g for 5 min at room temperature, the supernatant was discarded and cells were mixed with an equal volume of dip buffer (1 ml of 1 M MgCl_2_, 1 ml of 1 M MES, and 100 μl of 200 mM acetylbutyrophenone dissolved in 100 ml ddH_2_O) (OD600 = 1.0). Finally, cells were incubated for 2–4 h at room temperature.

We used the non-insect-resistant cotton ICR49 as the test plant. For the transformation of cotton, the epidermis on the abaxial surface of a cotton leaf was scratched with an insect dissection needle, and the *Agrobacterium* solution was injected through the wound. The transformed cotton plant was incubated for 24 h protected from light (25 ± 1°C and 60% ± 2% relative humidity). The cotton plant was allowed to grow to the 6-leaf stage and used to test the adaptability of cotton aphids.

### 2.6 Bioassay

Twenty adult aphids were transferred to VIGS cotton leaves as a group, and a miniature insect cage was fixed on the leaves to prevent aphids from escaping. After the adult aphids produced 25 T1 generation aphids, the adult aphids were removed. Seven days later, the average insect weight and the number of offspring were recorded, and the T1 generation cotton aphids were collected for RT-qPCR to determine the interference efficiency. Three biological replicates were set up in the experiment, with six groups in each biological replicate.

### 2.7 Statistical analysis

The significance of differences was analyzed using the Tukey’s HSD test with SPSS 20.0 (Chicago, IL, USA). *P* < 0.05 denotes a significant difference.

## 3 Results

### 3.1 Sequence analysis of *AgoArmet* and *AgoC002*

**Figure 2:**
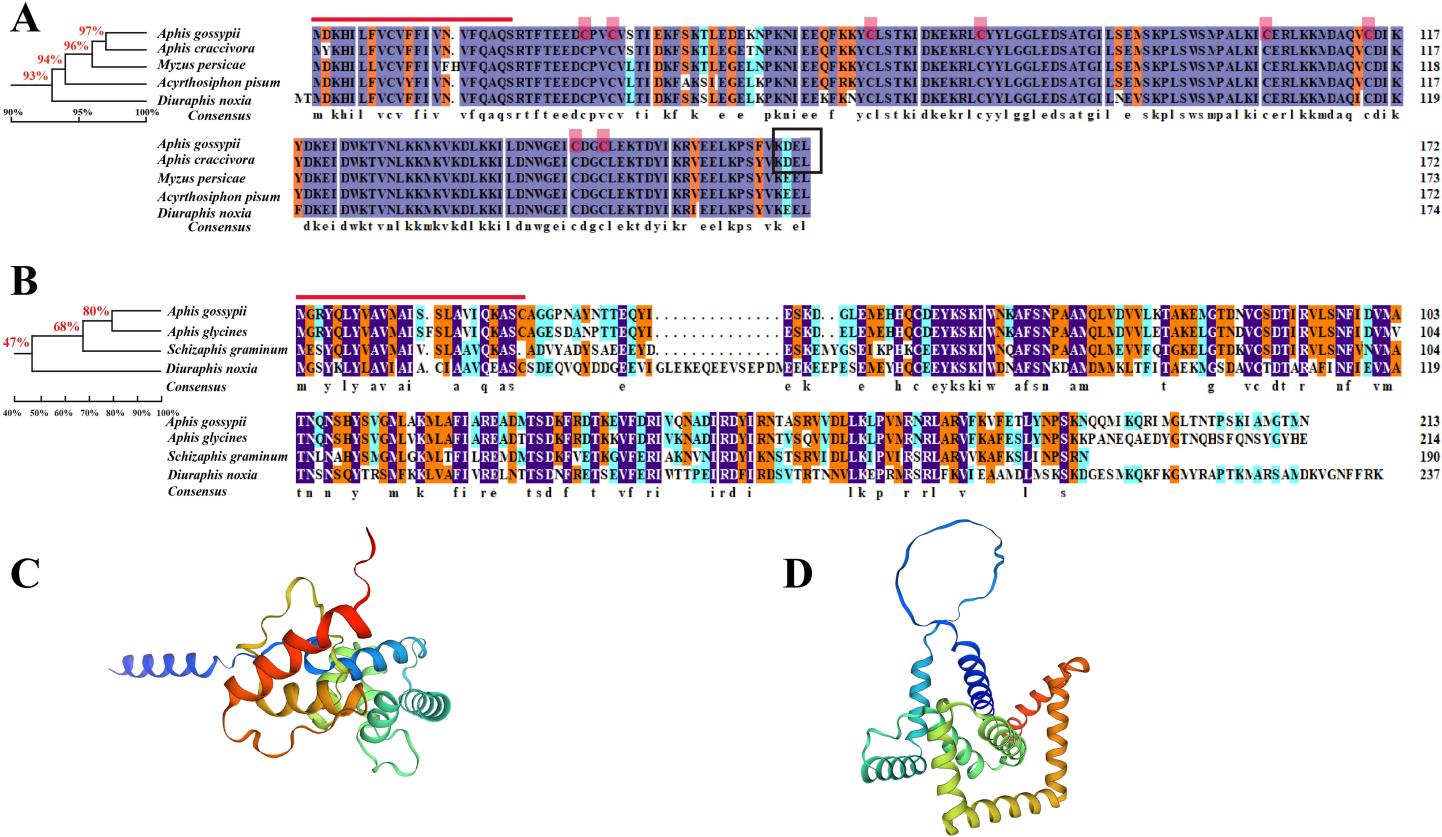
Amino acid sequence analysis of AgoArmet and AgoC002. Multiple sequence alignments and phylogenetic trees of Armet **(A)** and C002 **(B)** proteins from **(*A*)** *gossypii* and other aphids. The red line indicates the signal peptide, the red boxes indicate the cysteine sites, and the black box indicates the endoplasmic reticulum retention motif. The tertiary structures of the AgoArmet **(C)** and AgoC002 **(D)** proteins were constructed using SWISS-MODEL.

We performed multiple sequence alignment and protein structure prediction using the amino acid sequences of *AgoArmet* (NCBI GeneBank accession number: KY612465) and *AgoC002* (Accession number: AHX71991.1). The *AgoArmet* open reading frame (ORF) is 522 bp and encodes a protein of 173 amino acids, with a putative molecular mass of 20.3 kD, an isoelectric point of 6.6 (**Table 2**), and eight conserved cysteine sites (**Figure 2A**). A signal peptide of 21 amino acids was predicted by SignalP5.0, and the ER retention motif KDEL was identified at the C-terminus of the protein (**Figure 2A**). We selected the amino acid sequences of the *Armet* genes of *Aphis craccivora* (GenBank accession number: KAF0756441.1), *M*. *persicae* (GenBank accession number: XP_022167424.1), *A*. *pisum* (GenBank accession number: XP_001949541.1), and *Diuraphis noxia* (GenBank accession number: XP_015365890.1) for multiple sequence alignment with the cotton aphids proteins. The highest amino acid sequence identity was between the *A*. *pisum* and cotton aphids Armet proteins (97%). Sequence analysis indicated that AgoArmet was highly conserved during the evolution of insects (**Figure 2A**).

**Table 2:** AgoArmet and AgoC002 sequence information.

Gene ID	ORF (bp)	Number of amino acid	Relative molecular mass (kDa)	pI
AgoArmet	522	173	20.3	6.6
AgoC002	747	213	24.1	9.1

*AgoC002* has a full-length ORF of 641 bp, encoding a 213 amino acid protein with an estimated molecular mass of 24.1 kD and an isoelectric point of 9.1 (**Table 2**). It has a predicted signal peptide of 24 amino acids (**Figure 2B**). We compared the amino acid sequence of *AgoC002* with those of C002 proteins from *Aphis glycines* (GenBank Accession Number: AEV66513.1), *Schizaphis graminum* (GenBank Accession Number: AHN09735.1), and *D*. *noxia* (GenBank Accession Number: AEP03182.1). The highest amino acid identity was observed between the *A*. *glycines* and cotton aphids C002 proteins (80%) (**Figure 2B**). **Figures 2C**, **D** showed the protein structure of AgoArmet and AgoC002, respectively.

Phylogenetic analysis showed that AgoArmet and AgoC002 belong to different branches and are more distantly evolutionarily related between Armet and C002. AgoArmet is more closely related to Armet of Aphididae, and more distantly belonged to a different branching of other insects (**Figure 1**). We found the closest evolutionary relationship to AgoArmet to be *A*. *craccivora* C002, followed by *Melanaphis sacchari*. AgoC002 is phylogenetically most closely related to *A*. *glycines* C002, followed by *S*. *graminum* C002 and *D*. *noxia* C002 (**Figure 1**).

### 3.2 Gene expression profiles of *AgoArmet* and *AgoC002*

**Figure 3:**
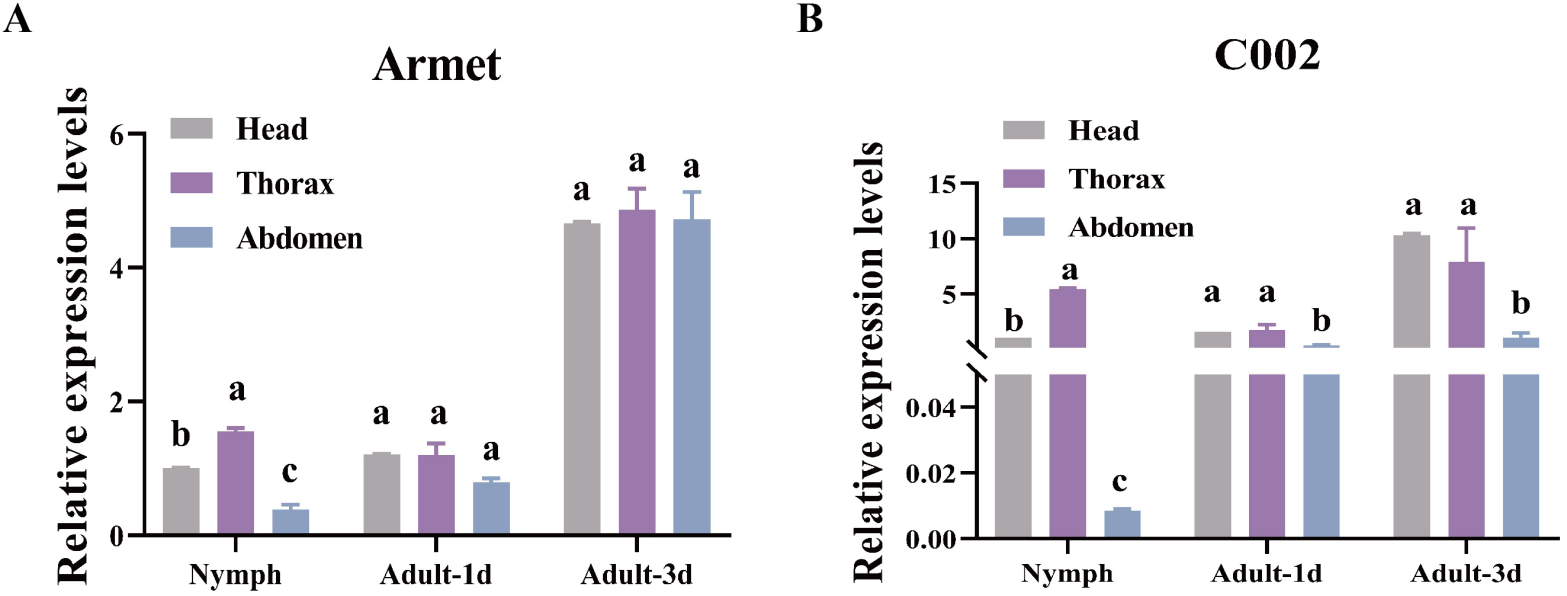
*AgoArmet* and *AgoC002* expression profiles. We examined the expression of *AgoArmet* **(A)** and *AgoC002* **(B)** in the head, thorax, and abdomen at different developmental periods. Different letters indicate significant differences according to Tukey’s HSD test (*P* < 0.05).

We explored the gene expression profiles of *AgoArmet* and *AgoC002* in different tissues (head, thorax, and abdomen) during the nymph, adult-1d, and adult-3d periods using RT-qPCR. *AgoArmet* expression was highest during the adult-3d period; there was no significant difference in expression between the head, thorax, and abdomen during the adult-1d or -3d period (**Figure 3A**). During the nymph period, the highest expression of *AgoArmet* was observed in the thorax and the lowest expression was observed in the abdomen (**Figure 3A**). During the nymph period, the expression of *AgoC002* in the thorax was significantly higher than that in the head and abdomen. During the adult-1d phase, there was no significant difference in *AgoC002* expression across tissues; however, during the adult-3d period, the head had the highest level of *AgoC002* expression (**Figure 3B**).

**Figure 4:**
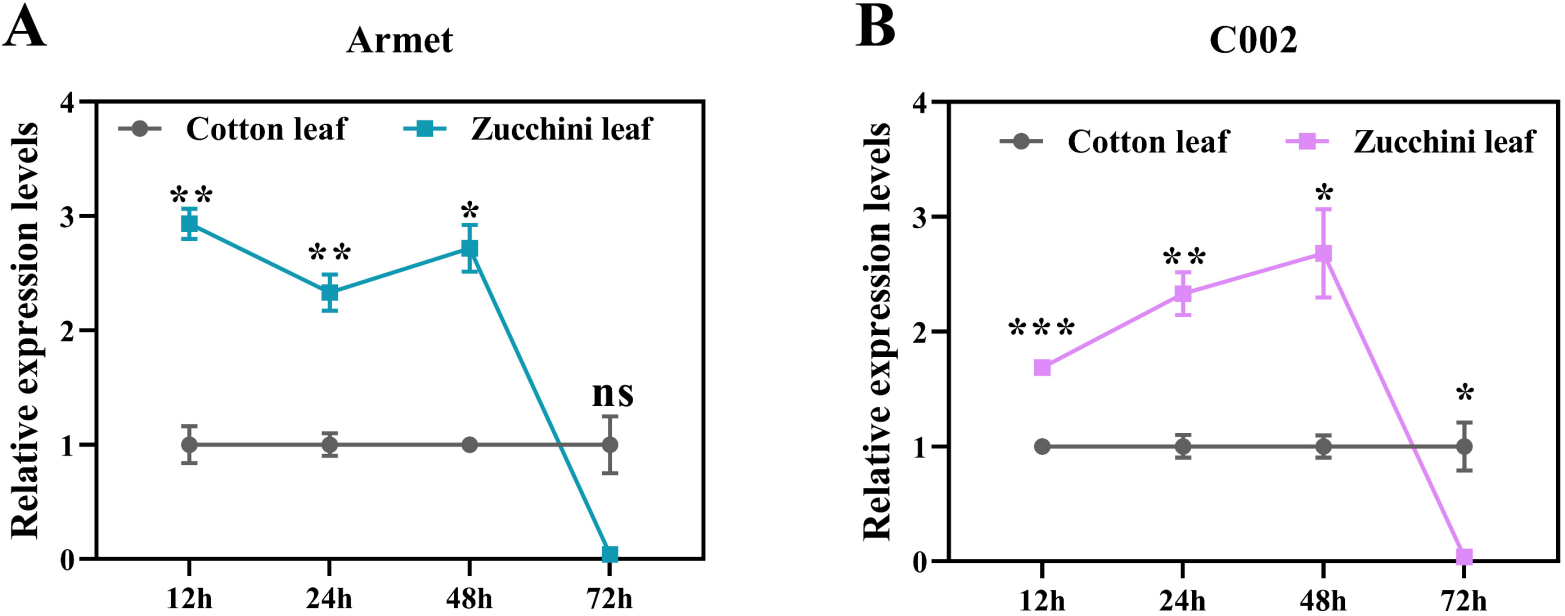
Effect of cotton aphids transfer to zucchini leaves on the expression of **(A)** *AgoArmet* and **(B)** *AgoC002*. Student's *t*-test was used for significance analysis (ns, not significant, *0.01 < p < 0.05, **0.001 < p < 0.01, and ***p < 0.001).

Zucchini is an important vegetable host for *A. gossypii*, and we found that the expression of *AgoArmet* and *AgoC002* was significantly increased at 12h, 24h, and 48h after *A. gossypii* was transplaced on zucchini leaves with cotton as the host (**Figures 4A, B**). However, the expression of *AgoArmet* and *AgoC002* of *A. gossypii* was decreased 72h after transplants to zucchini (**Figures 4A, B**).

### 3.3 VIGS-induced knockdown of *AgoArmet* and *AgoC002* gene expression in the host reduces the fitness of cotton aphids

**Figure 5:**
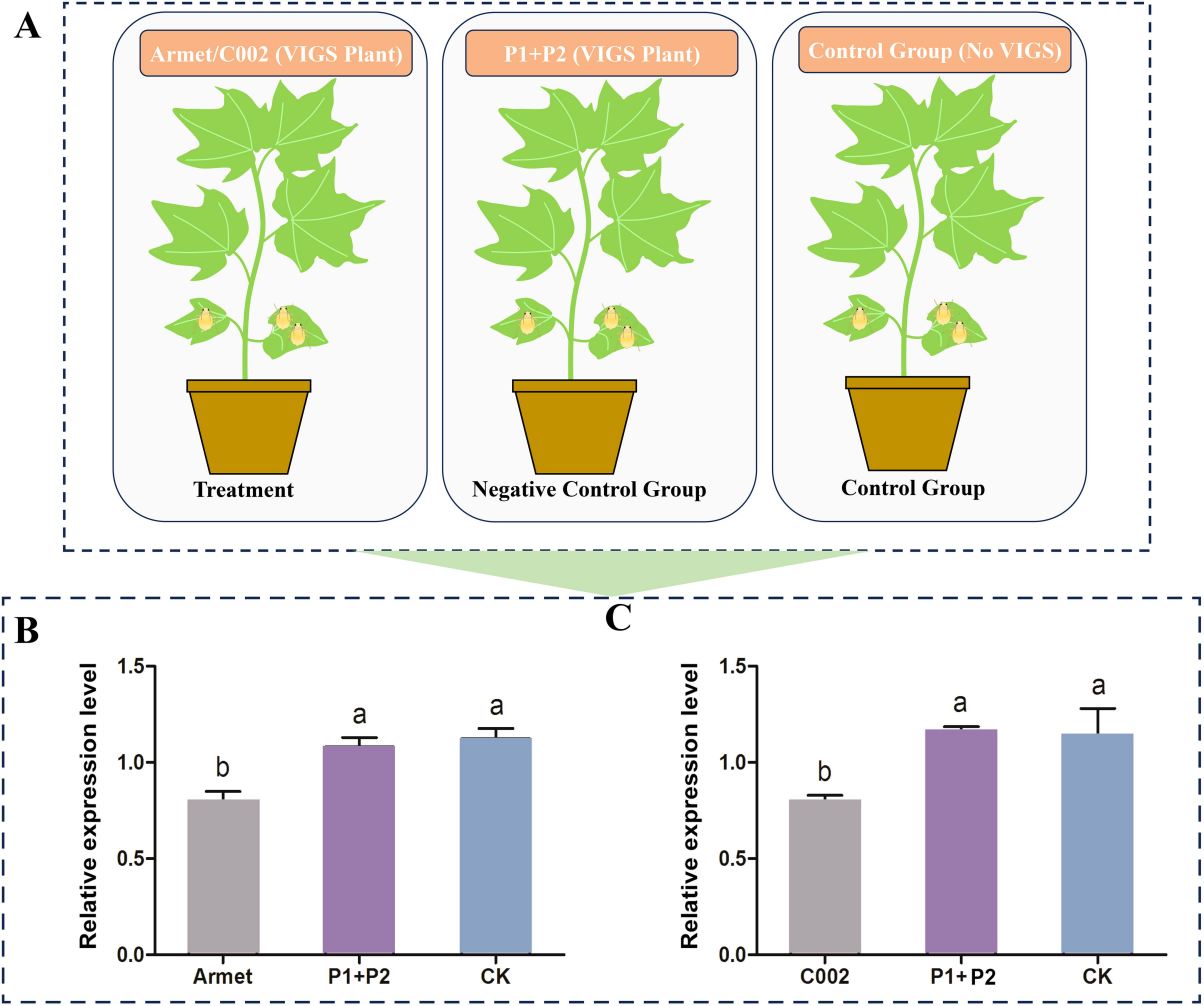
Detection of target gene silencing efficiency in VIGS plants. **(A)** Experimental groups and treatments. Cotton plants were transfected with vectors for knockdown of *AgoArmet* **(B)** and *AgoC002* **(C)** expression using virus-induced gene silencing (VIGS). Armet and C002 represent cotton plants transfected with target gene disruptors, P1+P2 represent cotton plants transfected with empty vectors (pTRV1+ pTRV2), and CK represents wild-type cotton plants. Different letters indicate significant differences according to Tukey’s HSD test (*P* < 0.05).

To determine the effect of knocking down the expression of *AgoArmet* and *AgoC002* on cotton aphids, we transfected cotton plants with *AgoArmet* and *AgoC002* disruptors using VIGS technology; cotton plants transfected with the empty vectors (pTRV1+ pTRV2) were used as a negative control (**Figure 5A**). Expression levels of *AgoArmet* and *AgoC002* in cotton aphids feeding on trans-*AgoArmet* and -*AgoC002* cotton leaves were significantly lower than those in aphids feeding on trans-empty vector and wild-type cotton plants (**Figures 5B, C**). Compared with cotton aphids feeding on the wild-type cotton plants, those feeding on cotton plants transfected with *AgoArmet* and *AgoC002* disruptors showed a 22% and 21% decrease in *AgoArmet* and *AgoC002* expression, respectively, indicating that the target genes were silenced.

**Figure 6:**
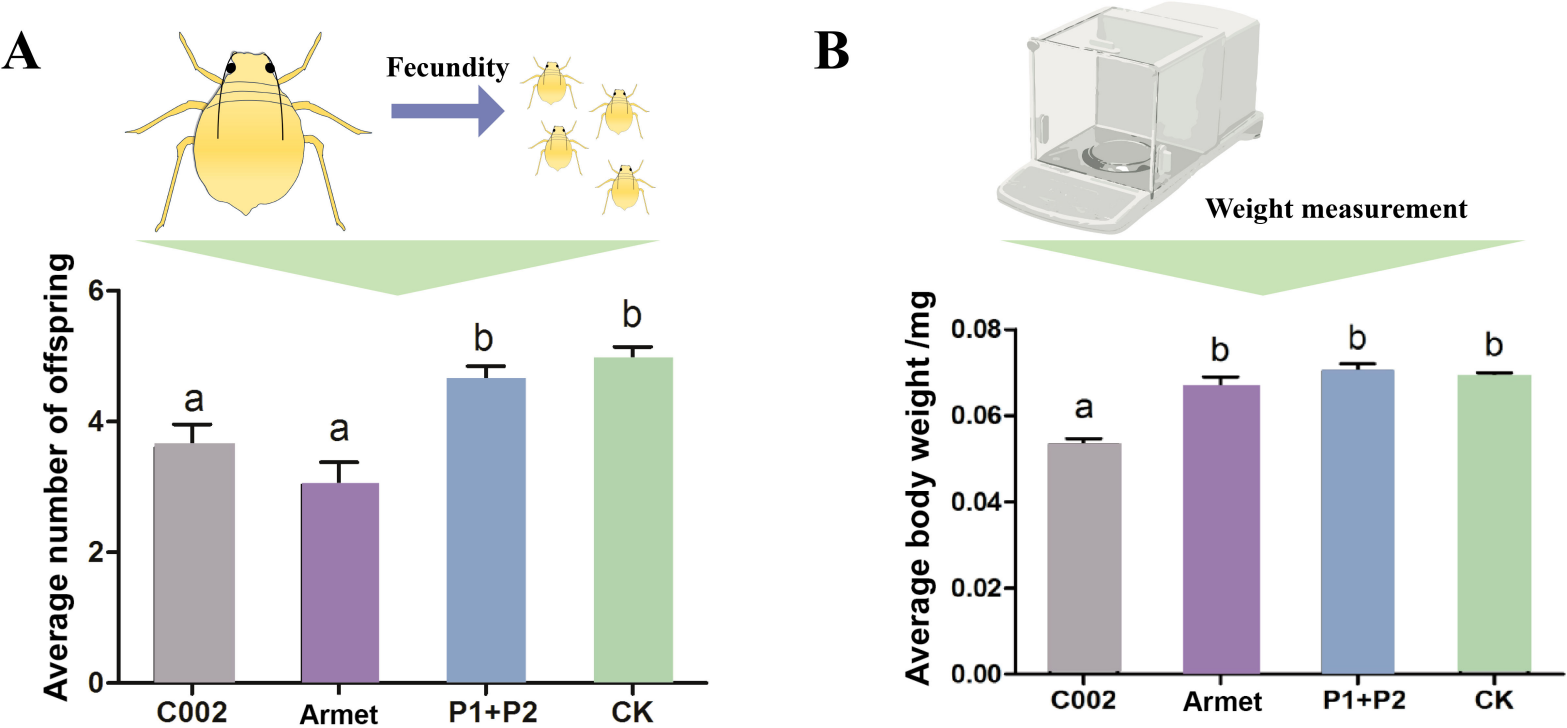
Functional analysis of AgoArmet and AgoC002. Changes in the number of cotton aphids offspring **(A)** and body weight **(B)** were recorded after VIGS knockdown of *AgoArmet* and *AgoC002* expression. Armet and C002 represent cotton plants transfected with target gene disruptors, P1+P2 represent cotton plants transfected with empty vectors (pTRV1+ pTRV2), and CK represents wild-type cotton plants. Different letters indicate significant differences according to Tukey’s HSD test (*P* < 0.05).

To evaluate the importance of these two effector genes for host adaptation in cotton aphids, we recorded the fecundity and body weight of cotton aphids feeding on cotton plants transfected with *AgoArmet* and *AgoC002* disruptors, negative control cotton plants transfected with empty vectors, and wild-type cotton plants. We found that the number of cotton aphids offspring was significantly lower in the *AgoC002* and *AgoArmet* silencing treatment groups, with a 26% and 38% reduction in the number of offspring observed, respectively, compared with the wild-type group (**Figure 6A**). We also found that reduced expression of *AgoC002* resulted in a significant (24%) decrease in cotton aphids body weight compared with the wild-type group (**Figure 6B**). However, knockdown of *AgoArmet* expression had no significant effect on the body weight of cotton aphids. These findings suggest that AgoArmet and AgoC002 play very important roles in the feeding and host adaptation of cotton aphids.

## 4 Discussion

Saliva serves as a medium for insect-plant interactions, and salivary proteins, which are the major effector molecules, are important weapons used by phloem-feeding insects to overcome plant defenses and facilitate feeding (Wang et al., 2015; Chen and Mao, 2020). In this study, we identified two genes encoding the salivary effector proteins Armet and C002 in the *A. gossypii* genome, and we found that these proteins belong to two different evolutionary branches (**Figure 1**). The expression levels of *AgoArmet* and *AgoC002* were the highest during the 3d-adult period (**Figure 3**). In addition, functional analysis confirmed that these two salivary effector proteins play an important role in the host adaptation of cotton aphids. Reduced expression of *AgoArmet* and *AgoC002* significantly reduced aphid fecundity and reduced expression of *AgoC002* significantly reduced body weight, suggesting that growth performance of the aphid on the host plant was reduced.

Multiple sequence alignment showed that AgoArmet is closely related to Armet from other insects with very high sequence identity, which can demonstrate that conservative Armet acts phylogenetically and evolutionarily on the feeding habits of cotton aphids. Armet has been reported to be an effector protein that plays a key role in the feeding behavior and growth performance of *A*. *pisum* on host plants (Wang et al., 2015). Our results showed that the function of AgoArmet in cotton aphids is also relevant to the promotion of feeding and host adaptation. Down-regulation of *AgoArmet* expression in cotton aphids significantly reduced their fecundity. This be explained by the close relationship between insect fecundity and nutrition and the fact that insect feeding is a key pathway for obtaining nutrients; weakening feeding will inevitably lead to an insufficient supply of nutrients (Roy et al., 2018). Another reason for reduced fecundity may be related to the defense response by the host plant. Plant defense responses to aphid feeding typically involve induction of the SA, JA, and ethylene signaling pathways (Zhang et al., 2020; Liu et al., 2021; Gao et al., 2023). Aphids must secrete biologically active effector proteins into the host plant to modulate the plant’s defense response and enable successful feeding (Waksman et al., 2024). The effect of reduced *Armet* expression in the host plant is a decrease in the production of salivary Armet by the aphid. In early annotations of the *A*. *pisum* genome, it was predicted that Armet has calcium-binding capacity and that it inhibits host plant defense responses by binding Ca^2+^ in the plant and preventing the host plant from initiating a response (Will et al., 2007; Du et al., 2022). Inhibition of plant defense responses by aphids is critically linked to increased aphid fecundity, and silencing of the salivary effector molecule Sm9723 in *Sitobion miscanthi* significantly reduces fecundity and survival as well as adversely affects feeding behavior (Zhang et al., 2022b). Although cucumber and zucchini are also important hosts for the cotton aphids (Zhang et al., 2022a), changes in hosts can also challenge the aphid’s physiological metabolism (Yates and Michel, 2018). Overexpression of the salivary protein Mp55 from *M. persicae* in Arabidopsis also significantly increased aphid colonization (Elzinga et al., 2014). Elevated expression of AgoArmet enhances the adaptation of cotton aphids to cucumber and zucchini hosts (**Figure 4A**), and this explanation also applies to AgoC002 (**Figure 4B**). Thus, salivary proteins may increase cotton aphids adaptations to plants by promoting feeding or resisting plant defenses.

C002 has been identified as another important insect salivary protein effector that can inhibit plant defenses and thereby reduce host plant susceptibility to invaders (Chaudhary et al., 2015; Fu et al., 2023). C002 was shown to be highly expressed in aphid salivary glands (Mutti et al., 2006), which are located in the head and anterior part of the thorax. Here, we found that *AgoC002* is very highly expressed in the head and thorax of the cotton aphids at different stages of development. Pitino et al. found that *M*. *persicae* feeding on *Arabidopsis thaliana* and tobacco plants transformed with the *dsMpC002* gene, had significantly fewer progeny and significantly lower expression of the *MPC002* gene (Pitino et al., 2011). The C002 effector protein is also critical for *M*. *persicae* colonization on host plants, and silencing of *C002* led to a reduction in *M*. *persicae* populations (Pitino and Hogenhout, 2013). Consistent with these findings, we also found that AgoC002 plays a critical role in host feeding and colonization of cotton aphids, and that silencing *AgoC002* led to significant reductions in cotton aphids fecundity and body weight, as well as a decrease in cotton aphids populations. The reduction in cotton aphids body weight is also a manifestation of plant defense against insects; plant defense compounds may repel or poison insects, and defense proteins usually interfere with digestion, leading to reduced body weight (Fürstenberg-Hägg et al., 2013).

To date, studies of Armet and C002 have mainly focused on *A*. *pisum* and *M*. *persicae*, and research on these proteins in cotton aphids, a major pest worldwide, is lacking. Because of the diversity of hosts and the wide distribution of aphids, there has been rapid evolution of genes related to adaptation (Ollivier et al., 2010; Jiang et al., 2023). Therefore, it is also very important to study the adaptation of cotton aphids to their hosts. Our results demonstrated the important role key salivary effector proteins of cotton aphids for feeding, and these results provide an important experimental basis for the future study of the mechanism of the interactions between cotton aphids and host defense responses.

In summary, our study identified two important salivary proteins of cotton aphids, AgoArmet and AgoC002, which play key roles in host plant feeding and plant defense. Reducing the expression of these two genes led to a decrease in the performance of the cotton aphids on the host plant and significantly reduced its fecundity. The down-regulation of *AgoC002* also led to a decrease in aphid body weight. These findings reveal an important molecular mechanism underlying the trade-off between plant defense and insect counter-defense. They also provide an important theoretical basis for the control of cotton aphids. It also provides new molecular targets to guide the green prevention and control of agricultural pests.

## Data Availability

The original contributions presented in the study are included in the article/supplementary material, further inquiries can be directed to the corresponding authors.
